# A rare case of IgG4-related disease: a gastric mass, associated with regional lymphadenopathy

**DOI:** 10.1186/s12893-016-0151-4

**Published:** 2016-06-02

**Authors:** Dimitar Bulanov, Elena Arabadzhieva, Sasho Bonev, Atanas Yonkov, Diana Kyoseva, Tihomir Dikov, Violeta Dimitrova

**Affiliations:** Department of General and Hepato-pancreatic Surgery, Medical University of Sofia, University Hospital “Alexandrovska”, 1 Georgi Sofiiski Str, 1431 Sofia, Bulgaria; Department of General and Clinical Pathology, Medical University of Sofia, 2 Zdrave Str, 1431 Sofia, Bulgaria

**Keywords:** IgG4-related disease, Stomach, Plasma cells, Pseudotumor, Surgical treatment

## Abstract

**Background:**

IgG4-related disease (IgG4-RD) is a newly recognized disorder, characterized by massive IgG4+ lymphocyte and plasma cell infiltration, storiform fibrosis, causing enlargement, nodules or thickening of the various organs, simultaneously or metachronously. Involvement of the gastrointestinal tract is very rare and can be presented as a diffuse wall thickening or polyp or mass-like lesion. Up to now, there have been reported only a few cases of isolated gastric IgG4-RD.

**Case presentation:**

We present an unusual case of IgG4-RD of the stomach with involvement of the regional lymph nodes, clinically manifested as a gastric cancer with related pyloric stenosis. The patient underwent distal gastrectomy, omentectomy and lymph node dissection. The postoperative serum IgG4 level was increased. The diagnosis was confirmed by immunohistochemical study.

**Conclusions:**

In the most of the reported cases there was not sufficient data about the regional lymph nodal status, although the majority of the patients had been operated with presumptive diagnosis of gastric neoplasm. Our case is rare and valuable because it presents a gastric IgG4-related lesion larger than all previously reported in literature, and IgG4-related lymphadenopathy, confirmed histologically, which contributes to better knowledge of the disease.

## Background

IgG4-related disease (IgG4-RD) is a newly recognized disorder, characterized by massive IgG4+ lymphocyte and plasma cell infiltration, storiform fibrosis, causing enlargement, nodules or thickening of the various organs, simultaneously or metachronously [1–4]. Other typical features are obliterative phlebitis, increased IgG4-expressing plasma cells, and often, but not always elevated serum IgG4 level [2, 3]. IgG4-RD frequently might be misinterpreted clinically and radiologically as a neoplasm, resulting in overtreatment [1–4]. The disease usually affects the pancreas, bile ducts, gallbladder, liver, lacrimal glands, salivary glands, retroperitoneum and lymph nodes [1–4]. Involvement of the gastrointestinal tract is very rare and can be presented as a diffuse wall thickening or polyp or mass-like lesion [3, 4]. Up to date, there have been reported only a few cases of isolated gastric IgG4-RD [3].

## Case presentation

We described the case of suspected gastric IgG4-RD and regional lymphadenopathy, misdiagnosed as a gastric cancer. A 62-year-old female presented with symptoms of severe weakness and fatigue. Laboratory tests revealed anemia (hemoglobin 58 g/L). Because of these the patient had been admitted to another hospital where a gastric tumor and related pyloric stenosis were found with gastroscopy and several blood transfusions were performed. The biopsy showed a chronic ulcerative lesion with suspected atypical cells in the bottom. Computer tomography (CT) revealed prepyloric irregular thickening of the gastric wall up to 15 mm which involved its entire circumference. The patient’s co-morbidities included Henoch-Schonlein purpura, allergy to analgesics, arterial hypertension and depressive disorder. Several years ago, the patient had undergone cholecystectomy and plastic of hiatal hernia. The physical examination of the abdomen did not reveal any pathological findings. Given the presumptive diagnosis of gastric cancer, the patient was hospitalized in our department for surgical treatment. A distal gastrectomy, omentectomy and lymph node dissection were performed. The diagnosis was confirmed with histological and immunohistochemical study.

**Fig. 1:**
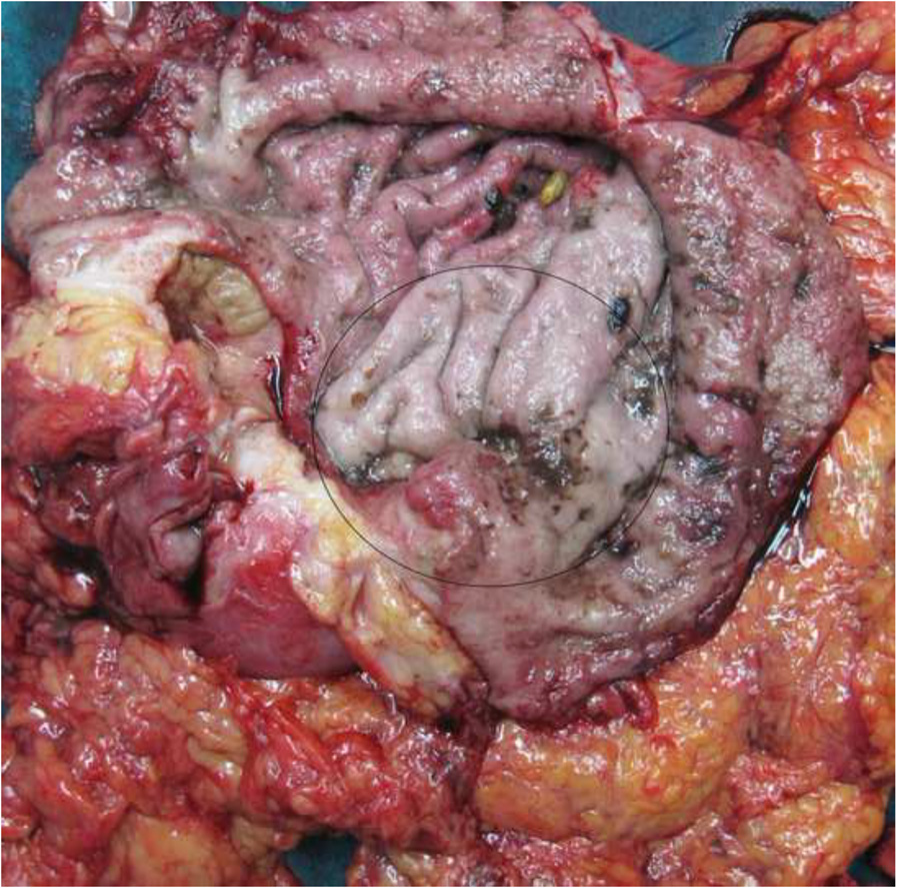
Macroscopic view of the gastric specimen – transected stomach with presence of an ulcer-infiltrative tumor formation, marked with a circle

Grossly, there was an ulcer-infiltrative tumor formation, 8/3 cm in size, involving gastric lesser curvature, the front and posterior wall of the stomach. The tumor infiltrated the lesser omentum and penetration into the serosa was probable. The surrounding mucosa was scattered with petechial hemorrhages (Fig. 1).

**Fig. 2:**
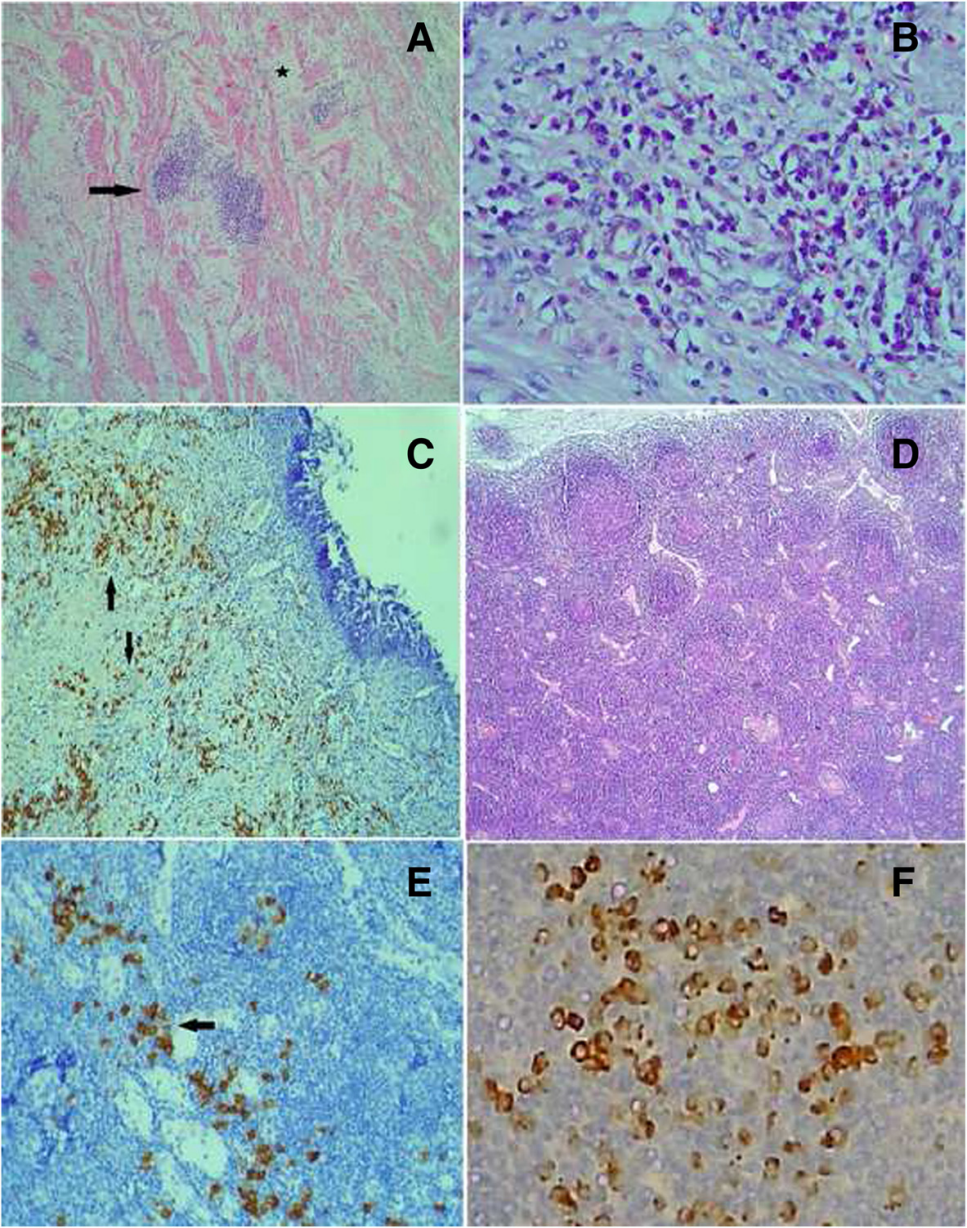
Histologic findings in the stomach and regional lymph nodes: **a** The gastric muscle layer with inflammatory infiltration from lymphocytes and plasma cells (*marked with an arrow*) and concomitant stroma dividing the muscle structures (*an asterisk*), H&E x4; **b** Detailed cellular composition, H&E x40; **c** abundance of CD 138+ plasma cells in area of the gastric lesion (*shown with arrows*); **d** Microscopic changes in lymph nodes, H&E x4; **e** Lymph node with interfollicular CD 138+ plasma cells, marked with an arrow; **f** Presence of IgG4-positive plasma cells

Microscopic examination of the gastric formation showed tumor-like proliferation with mixed cellular composition: predominantly CD79a + mature B-cells, organized in follicular structures and located diffusely; abundance of mature CD 138+ plasma cells and significant presence of eosinophils (Fig. 2 a, b and c). There was marked fibrosis in lamina propria, often in a storiform pattern of growth, induced by the lymphocyte and plasma cell infiltration. Proliferation of vessels, the same cellular infiltration and obliterative phlebitis were found, too. Sixteen lymph nodes were isolated. Histologically, they were characterized by preserved capsule thickness, presence of lymph follicles in the entire area of the node with activation changes, expressed in different degrees, and predominantly regressive changes of the germinal centers - depletion of the normal composition, vascularization, compact FDC-nets, dominated by CD 23+ cells (Fig. 2d). There were groups and bundles of mature CD 138+ plasma cells in the interfollicular areas (Fig. 2e). Immunohistochemical study was performed and abundance of positive IgG4 plasma cells was proved (Fig. 2f). In the gastric lesion there were more than 50 IgG4-positive lymphoplasmacytic cells per high-powerfield.

The postoperative period was uneventful. Serum IgG4 level measured postoperatively was 193.1 mg/dL (reference values 23–111 mg/dL).

## Discussion

IgG4-RD was firstly described in patients with sclerosing cholangitis, called autoimmune pancreatitis (AIP) type I, and later observed in other organs [1]. The comprehensive diagnostic criteria for IgG4-RD include: 1/diffuse or partial enlargement, swelling, nodules, or thickening lesion on single or multiple organs, 2/a serum IgG4 concentration more than 135 mg/dL, 3/histological changes a/massive lymphocytic and plasmacytic infiltration and sclerosis, b/increased number of IgG4+ plasma cells; and IgG4+/IgG+ plasma cell ratio >40 % and > 10 cells per high-powered field. Patients fulfilling all three criteria can be diagnosed definitively with IgG4-RD, those who fulfill criteria 1 and 3 have a probable diagnosis, and in those who fulfill criteria 1 and 2 the diagnosis is possible [1, 5]. However, these criteria do not include gastric localization of the disease. So a recent study established that infiltration of IgG4+ plasma cells in the stomach mucosa only cannot be diagnosed as gastrointestinal lesion of IgG4-RD [4]. This diagnosis can be made if there is diffuse wall thickening or polyp or mass-like lesion with relevant histological changes [4]. According to this, we found several cases of gastric IgG4-RD, reported in literature [3, 6–13]. A summary of them is described in Table 1.

**Table 1:** Summary of the cases with gastric IgG4-RD, reported in the literature and our case

Case № [Ref. №]	Age/Gender	Location in stomach	Endoscopic finding	Size (mm)	Serum IgG4	Treat-ment	Associated diseases
1 [6]	58/M	Fundus and body	Nodule	14	Normal	Steroid	AIP, IgG4-related sialadenitis
2 [7]	74/M	Body	Multiple polyps		Increased	NA	AIP
3 [8]	75/F	Body	Polyp	56	Normal	WR	None
4 [9]	45/F	Fundus	Nodule	15	Normal	WR	Raynoud’s disease
5 [9]	60/M	Antrum	Multiple nodules	Up to 22	NA	DG	Autoimmune polyendocrinopathy
6 [10]	56/M	Body	Nodule	8	NA	ESR	Type 2 diabetes
7 [11]	59/F	Midbody	Mass	33	NA	WR	None
8 [11]	54/F	NA	Mass	21	Normal	WR	None
9 [3]	48/F	Midbody	Mass	36	NA	WR	None
10 [12]	55/F	Body	Nodule	20	Normal	ESR	Hashimoto’s thyroiditis, possible primary biliary cirrhosis
11 [13]	68/M	Antrum	Wall thickening	40	Increased	DG	IgG4-RD in lungs, skin and lymph nodes
12 [present case]	62/F	Antrum	Mass	80/30	Increased	DG	Henoch-Schonlein purpura, IgG4-RD in lymph nodes

*M* male, *F* female, *NA* not available, *WR* wedge resection, *DG* distal gastrectomy, *ESR* endoscopic submucosal resection, *AIP* autoimmune pancreatitis

The most gastric IgG4-RD was detected in middle- aged patients (45 to 75 years) with predominance of women, although the total number of patients is too small to reveal any meaningful data. The body of the stomach was affected mainly. The size was variable (8–56 mm in cases reported in literature) and tumor in our case was considerably larger than the other gastric lesions of IgG4-RD. In most of the cases the gastric lesion was solitary. The two patients with multiple polyps in the stomach also had AIP and autoimmune endocrinopathy, respectively. In 7 of all reported cases, there were associated autoimmune diseases. In four of these patients there was multi-organ involvement of IgG4-RD – one patient with concomitant AIP, one case – with AIP and IgG4-related sialadenitis, one patient with localization of the disease in stomach, lungs, skin and lymph nodes and our patient had lymphonodal involvement, too. The IgG4-related lymphadenopathy, as in our case, can be confirmed only by histological examination. According to criteria, classified by Sato et al. histological changes include: 1/Castleman’s disease-like morphology; 2/reactive follicular hyperplasia, 3/interfollicular plasmacytosis and immunoblastosis, 4/progressive transformation of germinal center-like, and 5/inflammatory pseudotumor-like morphology [1, 14].

In most of the reported cases of gastric IgG4-RD there was not sufficient data about the lymph nodal status, although they were treated surgically with presumptive diagnosis of gastric neoplasm. In our case the accurate lymph nodal dissection was performed and IgG4-related lymphadenopathy was confirmed immunohistochemically. No enlarged lymph nodes had been detected on CT and the dissection was carried out only because we had suspected oncological disease. So if we had not done it, we would have missed the involvement of lymph nodes of IgG4-RD.

The first therapeutic choice for management of IgG4-RD is the steroid treatment [1, 3]. However, most patients with gastric IgG4-RD were treated surgically, except one patient with concomitant AIP who underwent steroid therapy. This is probably because IgG4-RD frequently might be misinterpreted clinically and radiologically as a neoplasm [1–4]. In our case the surgery was necessary not only because of the suspected gastric cancer, but for the relevant pyloric stenosis.

## Conclusions

Most gastric IgG4-RD lesions are difficult to diagnose because of their rarity and it is of utmost importance to exclude neoplasm. Unnecessary surgery can be avoided if the IgG4-RD of the stomach is considered in the differential diagnosis. Our case is rare and valuable because it presents a gastric IgG4-related lesion larger than all previously reported in literature, and IgG4-related lymphadenopathy, confirmed histologically, which contributes to the better knowledge of the disease.

## Abbreviations

AIP, autoimmune pancreatitis; CT, Computer tomography; DG, distal gastrectomy; ESR, endoscopic submucosal resection; F, female; H&E, Hematoxylin and Eosin; IgG4-RD, IgG4-related disease; M, male; NA, not available; WR, wedge resection
